# Green Synthesis of Reduced Graphene Oxide Using the *Tinospora cordifolia* Plant Extract: Exploring Its Potential for Methylene Blue Dye Degradation and Antibacterial Activity

**DOI:** 10.1021/acsomega.4c00748

**Published:** 2024-04-24

**Authors:** Ravi Saini, Ranjeet Kumar Mishra, Pradeep Kumar

**Affiliations:** † Department of Chemical Engineering & Technology, Indian Institute of Technology (BHU), Varanasi 221005, Uttar Pradesh,India; ‡ Department of Chemical Engineering, Manipal Institute of Technology, Manipal Academy of Higher Education, Manipal, Karnataka 576104, India

## Abstract

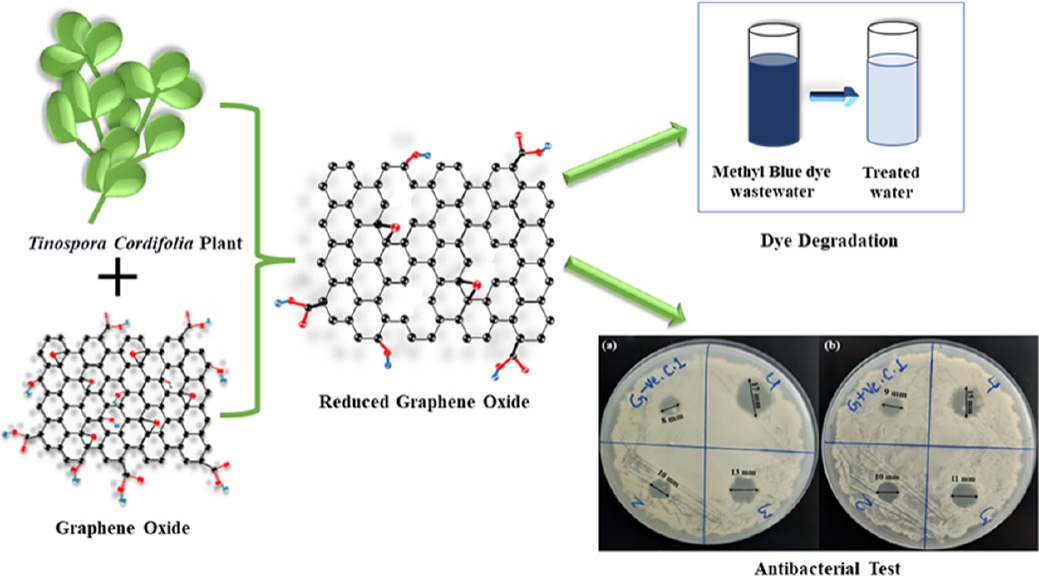

Graphene has attracted significant attention recently due to its unique mechanical, electrical, thermal, and optical properties. The present study focuses on synthesizing green rGO using the *Tinospora cordifolia* plant extract by mixing it in a suspension of graphene oxide. The plant extract of *T. cordifolia* acts as a reducing agent and is cost-effective, renewable, and eco-friendly. Green-synthesized rGO (G-rGO) was characterized using FTIR, HR-SEM, EDX, and HR-XRD analyses. G-rGO consists of nanosheets with an average width of approximately 30 nm. G-rGO has a range of hydrodynamic radius (270–470) nm and an average ζ potential of −29.9 mV. Further, G-rGO was used as a nanoadsorbent for optimal exclusion of methylene blue (MB) dye using the response surface methodology (RSM). Adsorption results confirmed 94.85% MB dye removal with 58.81 mg g^–1^ adsorption capacity at optimum conditions. The G-rGO’s antibacterial activity was also tested against *Staphylococcus aureus* (Gram-positive) and *Escherichia coli* (Gram-negative) bacteria, finding the exhibited zone of inhibition of 10, 11, and 15 mm and 10, 13, and 17 mm at 20, 40, and 80 μg mL^–1^ concentrations of G-rGO, respectively.

## 1 Introduction

Heterocyclic organic dyes are used in the cosmetic, culinary, plastic, leather, textile, paper, and pharmaceutical sectors. Methylene blue (MB) is also a heterocyclic dye and is the most commonly used dye.^1,2^ However, these sectors released an enormous volume of effluent into the surrounding environment that could have severe carcinogenic consequences, including *nausea, vomiting, diarrhea*, the development of the *Heinz bodies, jaundice, cyanosis, tissue necrosi*s, *quadriplegia*, disruption of the environment, and disturbances in the aquatic biodiversity. This calls for the complete and effective removal of MB dye.^2,3^ Consequently, a variety of techniques have been used to reduce the carcinogenic MB dye, including active carbon adsorption,^4^ dissolved air floatation,^5^ biochemical,^6^ chemical,^7^ and microorganism-mediated reductions.^8^ Even though the techniques mentioned earlier demonstrated concrete degradation activities, drawbacks such as their photolytic stability, pollutant phase transfer, high cost, and high resistance of the dye to microorganisms, the system still faces difficulty in removing the microorganisms from degraded dye molecules and preventing their widespread use.

Therefore, developing an adsorbent-based solution that is affordable, easy to recover from, and highly efficient in the degradation of MB dye is imperative, owing to its many active sites, excellent electron transfer capacity, high surface-to-volume ratio, high Fermi potential, superior manipulation, and high levels of thermal, chemical, and physical stability.

Nanoparticles (NPs) have recently captured the interest of the dye degradation field.^9^ Several nanoparticles, including Au, Ag, Pd, and Pt, have been widely used to accelerate the degradation of dye materials.^2^ The above-mentioned precious metal nanoparticles have several drawbacks, such as expensive cost, limited supply, and production of negative effects like eye discomfort, *argyria* (blue or blackish pigmentation), and skin allergy, which waned the desire for these valuable metal nanoparticles. Therefore, much attention has been paid to Fe, Cu, Ni, and Co (nonprecious metal nanoparticles) to use as catalysts for the degradation of the MB dye.^2^ The emphasis on Fe_3_O_4_ nanoparticles among nonprecious metal oxide nanoparticles is appealing because of their reactive surfaces, low cost, high specific area, faster magnetic separation after decomposition, reduced environmental pollution, and quickly oxidized surface.^10,11^ Fe_3_O_4_ nanoparticles quickly lose their active sites when reacting with the organic dye, causing high-speed nanoparticle agglomeration that cannot entirely break down the substance of the dye. The restrictions mentioned above might be overcome if the Fe_3_O_4_ NPs were adequately supported by active carbon. Graphene, carbon nanotubes, Vulcan carbon, and other carbonaceous nanomaterials are frequently used as supports to bind nanoparticles.^2^ The high electron transfer rate, tremendous surface area, better mechanical, electrical, charge carrier mobility, elastic behavior, and chemical and thermal characteristics of graphene make it much superior to the other carbon support listed above.^12^ With a high surface area and exceptional adsorption capabilities, graphene-based materials efficiently remove pollutants, such as heavy metals, organic compounds, and dyes, from water and air, contributing to environmental cleanup efforts. Moreover, their strong antibacterial properties make them effective agents against a wide range of pathogenic bacteria, offering potential solutions for combating bacterial infections and promoting public health. Chemical vapor deposition (CVD), solution-based chemical reduction, mechanical and ultrasonic exfoliation, epitaxial growth, electric arc discharge, and others are currently used to produce graphene on a large scale.^2,13−15^ These techniques have several limitations, such as the need for high temperatures and complex equipment, the production of lesser yields, and labor-, time-, and money-intensive characteristics. Because of this, a different approach based on the reduction of graphene oxide (GO) has been developed, and the resulting reduced graphene oxide (rGO) demonstrated higher conductivity (0.05 and 2 S cm^–1^), which is roughly three times higher magnitude than the GO and also comparable to graphene. Restoring a graphitic network of sp^2^ bonds is responsible for the increased surface area, effective carrier mobility, and improved electrocatalytic characteristics of rGO over GO.^16,17^ Primarily, powerful reducing chemicals such as dimethyl hydrazine,^12^ hydroquinone,^18^ and hydrazine hydrate^14^ are used to reduce GO. However, these above potent reducing agents showed some drawbacks, including the bonding of toxic elements and atoms of elemental nitrogen, the generation of toxic gases, and the partial elimination of oxygen-containing species, which endanger the environment and lower the effectiveness of rGO. Tin powder^19^ and iodide^20^ have additionally been utilized as efficient reducing agents for the total reduction of GO in addition to those mentioned above traditional reducing agents. The rGO possesses carboxyl groups (−COOH), carbonyl groups (C=O), hydroxyl groups (−OH), and epoxide groups (−O−), providing a diverse range of active sites for the adsorption of hydrophobic water pollutants. Tin substances have negative side effects, including thyroid gland tumors, genotoxicity, neurotoxicity, cutaneous toxicity, immunotoxicity, and hepatotoxicity. On the other hand, iodides can potentially have serious negative consequences on the human body, including hand numbness, pulmonary edema, *hyperthyroidism arrhythmia*, and heart failure.^2^ The major goal of the dye removal strategy is to reduce the degree of environmental toxicity, which cannot be achieved if the powerful reducing agents above enhance the toxicity of rGO sheets. Due to its eco-friendly methods, time- and cost-efficient features, the tendency for highly developed and intricate equipment, etc., renewable materials, particularly photosynthetic autotroph-mediated GO reduction, have attracted curiosity. Photosynthetic autotrophs can reduce metal ions in addition to GO sheets.

*T. cordifolia* stem extract was used to reduce GO and acts as a reducing agent. *T. cordifolia* is called Giloy; it is the most significant species within the Menispermaceae family. It is an herbaceous vine found in Sri Lanka, Myanmar, China, and India. Its aqueous extract is frequently used in medicine and is palatable. The *T. cordifolia* plant is abundantly available in the environment and does not require special planting conditions to grow independently. This plant extract has no environmental impact and is cost-effective compared with chemical-reducing agents. The extract of *T. cordifolia* contains various biochemicals, including alkaloids, glycosides, flavonoids, phenolic compounds, polysaccharides, and terpenoids, which can serve as reducing agents and stabilizers during the synthesis of rGO. The availability of *T. cordifolia* plants and seeds is missing in the literature. Several authors studied the reduction of GO to rGO using plant extracts and reported the findings in the literature. Eucalyptus leaves, ginger and garlic, espand seeds, and green tea extracts are used to prepare rGO, and it was reported that these extracts act as reducing and capping agents, and through various characterization, the green reduction of GO with improved characteristics like high electrical conductivity and excellent catalytic activity was confirmed.^21−24^ Kindalkar et al. used the *Syzygium samarangense* fruit extract for the green synthesis of rGO using Hammers methods. They stated an increased XRD peak position, increased *I*_D_/*I*_G_ ratio, high carbon-to-oxygen ratio, and red-shifted absorption peak.^25^ Finally, Yang et al. used the *Salvia spinosa* plant extract to synthesize rGO with many bioactive molecules. They stated that green-synthesized rGO has the potential to raise the temperature more than GO. Moreover, the nontoxic characteristic of RGO was demonstrated by a cell viability test performed using MTT dye.^26^ After reviewing several pieces of the literature, it was noticed that *Tinospora cordifolia* stem extracts are missing for synthesizing rGO and its application for Methylene Blue (MB) dye removal.

With the above-mentioned research gap and to the best of our knowledge, no research is available on the utilization of *T. cordifolia* plant stem extract as an antioxidant additive for reducing GO. The *T. cordifolia* stem extract is an eco-friendly, energy-efficient, and low-cost material used for the first time for the rGO synthesis to remove MB. Therefore, the present study focuses on the sustainable synthesis of rGO using *T. cordifolia* stem extract and application in removing MB. The response surface methodology (RSM) was used to analyze varied parameters like initial dye concentration, pH, catalyst dose, and adsorption time of the adsorption process. The effect of G-rGO’s antibacterial activity was also elucidated. Further, *Staphylococcus aureus* (Gram-positive) and *Escherichia coli* (Gram-negative) bacteria were used to investigate the bactericidal efficacy of bacterial pathogens. The feeds were characterized using XRD, FTIR, SEM, ζ-potential, particle size analysis, and UV–vis spectrometry. Table 1 explains a comparison of the current findings with existing research.

**Table 1:** Comparative Study of Dye Removal from Wastewater through Adsorption Using Plant Extracts to Synthesize an Adsorbent

s. no.	adsorbent	plant extract used	dye removed	% removal	refs
1	ZnO NPs	Lychee peel	Congo red (CR) dye	>98% in 2 h	(27)
2	CG-Zn(OH)_2_ NPs	*Calotropis gigantea* leaf	Coomassie violet (CV) dye	90.74% in 24 h	(28)
3	ZnS NPs	*Artemisia Herba Alba*	Methylene blue dye	94.09% in 3 h	(29)
4	ZnO NPs	Eucalyptus leaf	Acid black 210 dye	59% in 3 h	(30)
5	CuO@C nanocomposite	*Combretum indicum* plant	Malachite green, congo red, brilliant blue, and methylene blue	83.23, 84.60, 71.39, and 23.67% in 1.5 h	(31)
6	Ag NPs	*Ophiorrhiza mungos*	Methylene blue dye	88.11% in 1 h	(32)
7	Magnetic Fe_3_O_4_	*Cordia myxa* leaf	Methylene blue dye	88.8% in 1 h	(33)
8	G-rGO	*T. cordifolia*	Methylene blue (MB) dye	94.85 in 2.5 h	**present study**

## 2 Materials and Methodology

### 2.1 Materials

Pristine graphite powder (99% carbon, 325 mesh) and Methylene Blue dye were ordered from Sigma-Aldrich, India. All of the chemicals were used without treatment or purification in the experimental study and were analytical grade.

### 2.2 Extract Preparation of the *T. cordifolia* Plant Stem

*T. cordifolia* plant stems free of disease were collected, washed with distilled water several times, and then chopped and crushed. Further, 15 g of washed *T. cordifolia* stems was added in a 250 mL flask with 100 mL of distilled water and boiled for 3 h with constant stirring at 80–90 °C.^34,35^ The solution’s hue changed from colorless to brown and was cooled until it reached room temperature. The prepared solution was filtered by using Whatman filter paper (No. 1). The sample was collected in a glass vial and stored in the refrigerator at 0–5 °C for 1 week.

### 2.3 Preparation of GO

**Figure 1:**
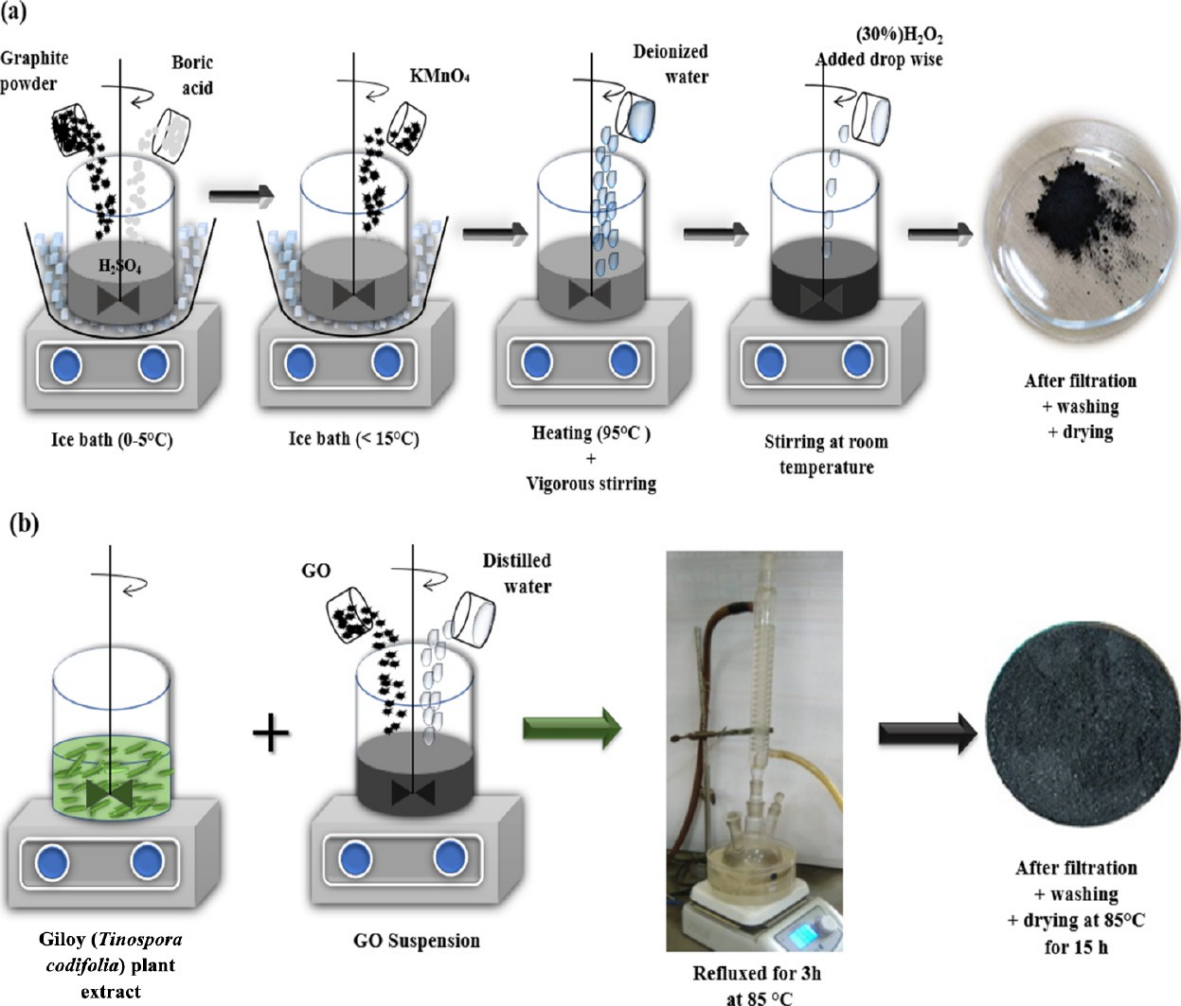
(a) Typical illustration of the formation of GO using modified Hummer’s method and (b) illustration of the synthesis of G-rGO from *T. cordifolia*.

GO was synthesized from natural graphite powder using modest tweaks to Hummer’s process (Figure 1a). A 5.0 g amount of dried graphite powder and 0.015 g of boric acid (H_3_BO_3_) were added to 120 mL of concentrated sulfuric acid (0.1 M H_2_SO_4_) in a 1000 mL flask held in an ice bath (0–5 °C). The solution was constantly stirred for roughly 3 h.^36^ The combination was placed in an ice bath (<15 °C to avoid explosion) for 2 h and 15 g of KMnO_4_ was slowly added. The solution was to be removed from the ice bath and heated to 35 °C for 2 h, stirring constantly. The stirred mixture was diluted with 450 mL of deionized water and heated to 95 °C for 2 h. Thereafter, the mixture was allowed to settle to ambient temperature for 1 h. Furthermore, 35 mL of hydrogen peroxide (H_2_O_2_, 30%) was added to eliminate KMnO_4_ residue. Finally, the solution was filtered (Whatman No. 1 filter paper, vacuum filtration), and the resulting precipitate was thoroughly rinsed with 500 mL of deionized water and dried in a hot air oven for 15 h at 85 °C.

### 2.4 Green Synthesis of rGO Using the *T. cordifolia* Stem Extract

A 50 mL solution of distilled water containing 25 mg of GO was sonicated for 1 h in a 100 mL Erlenmeyer flask. Further, an Erlenmeyer flask (250 mL) was used to combine 50 mL of *T. cordifolia* extract and 50 mL of GO suspension in 1:1 (v/v) ratio. The thoroughly mixed solution was refluxed at 85 °C for 3 h.^37^ Finally, the resulting solution was filtered (Whatman filter paper (No.1)), rinsed multiple times, and dried in an oven (85 °C) for 15 h. The dried G-rGO was ground into a fine powder for further experiments (Figure 1b).

### 2.5 Characterization of GO and G-rGO

The structural characteristics of GO and G-rGO were identified using a Rigaku SmartLab 9 kW Powder X-ray diffraction system with a Cu Kα radiation source (30 kV, 30 mA) keeping λ = 1.5406 nm. The range of 2θ used was 10–80° to obtain the diffraction patterns with 5° per minute scan rate. Further, SEM-EDX (MA15/18, Carl Zeiss microscopy equipped with Team Pegasus Integrated EDS-EBSD with octane plus and Hikari Pro) was used to display the images of the surface and elemental percentage of GO and G-rGO. A Malvern Panalytical, Zetasizer ver. 7.13 ζ potential analyzer was used to analyze the distribution of hydrodynamic particle sizes of GO and G-rGO and their ζ potential stability in colloidal solutions. The solution was prepared in 50 mL of distilled water by mixing 1 mg of GO and G-rGO powder. The FT-IR technique (Nicolet iS5, THERMO Electron Scientific Instruments LLC) was used to estimate the surface functional groups of GO, G-rGO, and *T. cordifolia* plant extract. An FTIR spectrophotometer was employed to obtain the spectra, which have spectral resolutions of 4 cm^–1^ step size of 1 s and an infrared wavelength range of 4000–500 cm^–1^. The absorbance of the aqueous MB dye solution at the λ_max_ (664 nm) was measured in a UV–vis spectrophotometer (SL-159, Elico, India) using a quartz cuvette to determine the sample concentration in the aqueous GO and G-rGO solution.

### 2.6 Batch Adsorption Experiments

The MB dye was removed by batch adsorption using G-rGO. An Erlenmeyer flask (250 mL) containing MB dye solutions (100 mL) was used for adsorption experiments. To maximize the MB dye’s adsorption, the effects of several variables were examined, including the initial pH of the solution, initial dye concentration, and duration of adsorption. Drop-by-drop additions of 0.1 M HCl and 0.1 M NaOH solution were used to change the solution’s initial pH. An incubator shaker (REMI, India) with temperature control was used to perform adsorption experiments. The range of temperature fluctuations in the incubator was 30 ± 5 °C with 130 rpm agitation speed. The following eqs 1 and 2 were employed to estimate the % removal of the MB dye and the adsorption capacity of the adsorbent, respectively^38^
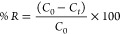
1
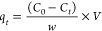
2where *C*_0_ and *C_t_* represent the initial concentration and concentration at any time *t* of the MB dye solution (mg L^–1^), respectively, *V* is the volume of the dye solution, *w* represents the amount of the adsorbent (g), % *R* represents the percentage removal of MB, and *q*_*t*_ represents the adsorption capacity of the adsorbent (mg g^–1^). The Langmuir and Freundlich isotherms were used for the adsorption isotherm study. The nonlinear forms of Langmuir and Freundlich isotherm models are shown in eqs 3 and 4, respectively
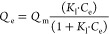
3
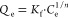
4where *Q*_m_ is the maximum adsorption capacity (mg g^–1^), *Q*_e_ is the amount of the adsorbate adsorbed per unit mass of the adsorbent (mg g^–1^), *K*_l_ is the Langmuir constant (L mg ^–1^), *C*_e_ is the equilibrium concentration of the residual MB dye (mg L^–1^), *K*_f_ is the Freundlich constant (L g^–1^) associated with the adsorption capacity, and  is the dimensionless heterogeneity factor.

### 2.7 Adsorption Kinetics

The adsorption kinetic experiment was performed by using the optimum conditions of parameters produced by the RSM model. In several conical flasks, the predicted initial dye concentration with the pH dye solution was prepared, and the optimum amount of the adsorbent was added to these flasks and then mixed thoroughly to ensure uniform mixing. Periodically, the solution was sampled from each flask for the next 250 min and the remaining dye concentration was measured using analytical techniques, such as UV–vis spectroscopy. The data of dye concentration versus time were plotted to analyze the adsorption kinetics.

### 2.8 Antibacterial Activity of G-rGO

The antibacterial activity of G-rGO was investigated against *Staphylococcus aureus* and *E. coli*. In this study, a sterile nutrient agar medium was prepared, cast into a Petri dish, and left for some time at room temperature to solidify. Subsequently, a 10 μL well-developed *S. aureus* and *E. coli*. culture was evenly distributed over the Petri dish and placed in a laminar airflow for 10 min. Further, 5–8 μL of G-rGO solution with different concentrations of 20, 40, and 80 μg mL^–1^ were dropwise placed over the bacteria with a 20 μg mL^–1^ solution of GO as a control on the Petri dish according to different marked places on the Petri dish. Antibacterial activity was examined after 12 h of incubation (30 ± 5 °C). Further, the zone of inhibition was calculated by measuring the circular diameter of the *S. aureus* and *E. coli* culture-free area on the Petri dish.

### 2.9 Optimization of Adsorption Using the Response Surface Methodology (RSM)

The RSM was used for optimization of the adsorption of the MB dye by using a central composite design (CCD) method. CCD was satisfactorily used to examine the impact of various factors on the effectiveness of the MB dye’s adsorption onto G-rGO. The ranges and levels of four independent variables were chosen: pH (A), G-rGO dose (B), initial dye concentration (C), and time (D) are mentioned in Table 2. The response of the system was defined by the percentage of MB dye removal. The optimal point prediction model based on a quadratic equation is expressed in eq 5
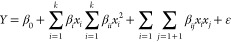
5where Y is the response (dependent variable), β_0_ is the constant coefficient, β_*i*_, β_*ii*_, and β_*ij*_, are the coefficients for the linear, quadratic, and interaction effects, respectively, *x*_*i*_ and *x*_*j*_ are the factors (independent variables), and ε is the error.

**Table 2:** Independent Process Variable Experimental Range and Levels

		Levels and ranges (coded)				
Independent variables	Designated symbol	–α	–1	0	+1	+α
pH	A	2.5	5	7.5	10	12.5
Adsorbent dose (mg)	B	5	20	35	50	65
Initial MB concentration (mg/L)	C	15	30	45	60	75
Time (min)	D	15	60	105	150	195

A standard RSM-based CCD was used to study the percentage removal of the MB dye. 30 experimental runs were carried out in triplicate by the specified scheme mentioned in Table 3. The data obtained were evaluated by graphical analysis and regression using Design Expert Version 13.0.5.0. The model’s fitness and results were examined using analysis of variance (ANOVA). The optimum values of the four independent parameters were determined by analyzing the response surface contour plots and solving the regression equation. The coefficients of multiple determination (*R*^2^) were used to explain the variability of the dependent variables, and the prediction of the optimum value and how the factors interacted with one another within a specified range was demonstrated by using the model equation.

**Table 3:** Central Composite Design Experimental Matrix

	pH	Adsorbent dose	Initial concentration	Time	% removal	
Run	A	B	C	D	Experimental	Predicted
1	7.5	5	45	105	79.67	78.64
2	7.5	65	45	105	94.87	94.42
3	7.5	35	75	105	88.51	87.39
4	7.5	35	45	105	90.89	89.93
5	7.5	35	45	105	88.32	89.93
6	5	50	60	60	85.15	87.45
7	7.5	35	15	105	67.47	67.11
8	12.5	35	45	105	64.92	65.29
9	10	20	60	150	75.15	75.49
10	10	20	60	60	69.63	70.42
11	5	20	30	150	50.15	51.41
12	10	50	30	60	89.85	89.76
13	10	50	30	150	95.9	96.44
14	5	20	30	60	41.25	41.84
15	7.5	35	45	105	90.58	89.93
16	5	50	30	60	61.2	60.48
17	5	20	60	150	90.33	92.28
18	7.5	35	45	15	72.54	73.15
19	5	50	30	150	67.35	68.43
20	10	20	30	60	73.91	73.88
21	5	20	60	60	86.86	85.93
22	10	50	60	150	71.35	72.63
23	10	20	30	150	82.6	82.17
24	10	50	60	60	70.83	69.18
25	2.5	35	45	105	54.65	52.80
26	5	50	60	150	92.53	92.18
27	7.5	35	45	105	89.82	89.93
28	7.5	35	45	195	88.27	86.18
29	7.5	35	45	105	90.07	89.93
30	7.5	35	45	105	89.92	89.93

## 3 Result and Discussions

### 3.1 Characterization of GO and G-rGO

#### 3.1.1 HR-XRD of GO and G-rGO

**Figure 2:**
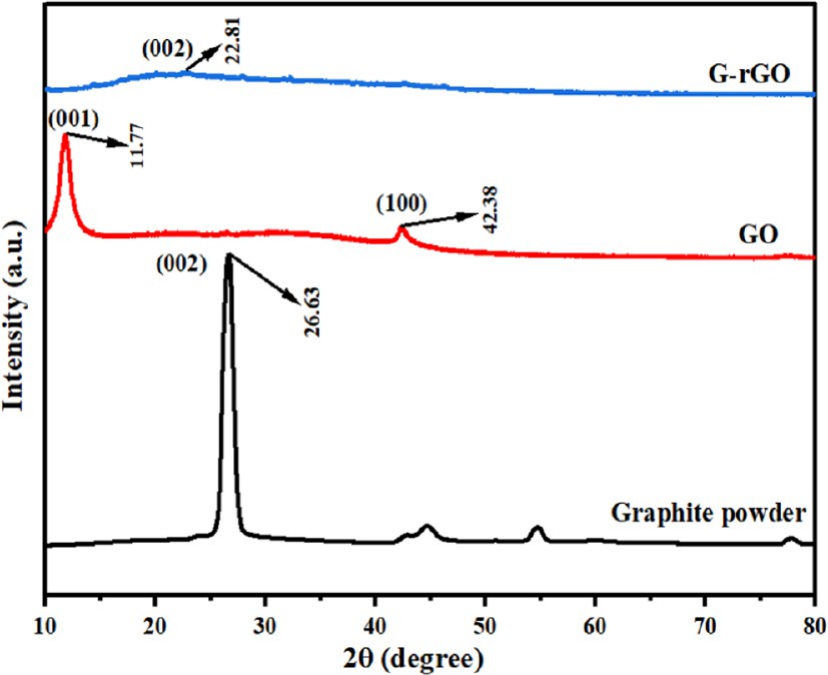
XRD spectra of graphite powder, GO, and G-rGO.

Powder X-ray diffraction analysis was carried out to validate the formation of the crystalline nature of the as-synthesized GO and the amorphous nature of G-rGO, listed in Figure 2. Bragg’s equation was used to determine the *d*-spacing values, which are described as *d* = *n*λ/2 sin θ, where d stands for the *d*-spacing or interplanar spacing, λ = 1.540 Å (wavelength of X-rays transmitted), and θ indicates the peak position (in Radians) with the order of diffraction (*n* = 1). Graphite, in its purest form, at 2θ = 26.63° had a sharp peak corresponding to layer structures that are well-organized along the orientation (002).^39^ Although Hummers’ method involved interactions with KMnO_4_ and H_2_SO_4_ (strong oxidants), this peak’s intensity and sharpness were reduced severely.^40^ Also, a new peak (001) at 2θ = 11.77° corresponding to functional groups that contain oxygen appeared, having a 0.751 nm *d*-space value. Compared to G-rGO, the peak disappeared because of the reduction of GO by the *T. cordifolia* stem extract. A less intense and broad reflection peak (002) was observed at 2θ = 22.81° for G-rGO with a 0.389 nm *d*-space value. The greater value of the *d*-space in GO indicated that O_2_ functional groups are formed, and water molecules intercalate between graphite layers. The decreased value of the *d*-spacing of G-rGO indicated that exfoliation had occurred more extensively because oxygen-related groups from the GO surface were removed.^41^ The reduction of GO includes the reduction or elimination of oxygenated functional groups on GO, resulting in the restoration of the sp^2^ carbon structure of graphene. The less intense and broader peak of G-rGO suggests a decrease in the crystallinity and long-range order compared to the highly ordered structure of GO. The broadening of the peak indicated the transformation of GO into a more disordered and defective structure, providing insights into the reduction process and the resulting properties of G-rGO. The G-rGO’s XRD pattern mostly matched the previously reported graphene pattern.^42^

#### 3.1.2 FTIR Analysis of GO and G-rGO

**Figure 3:**
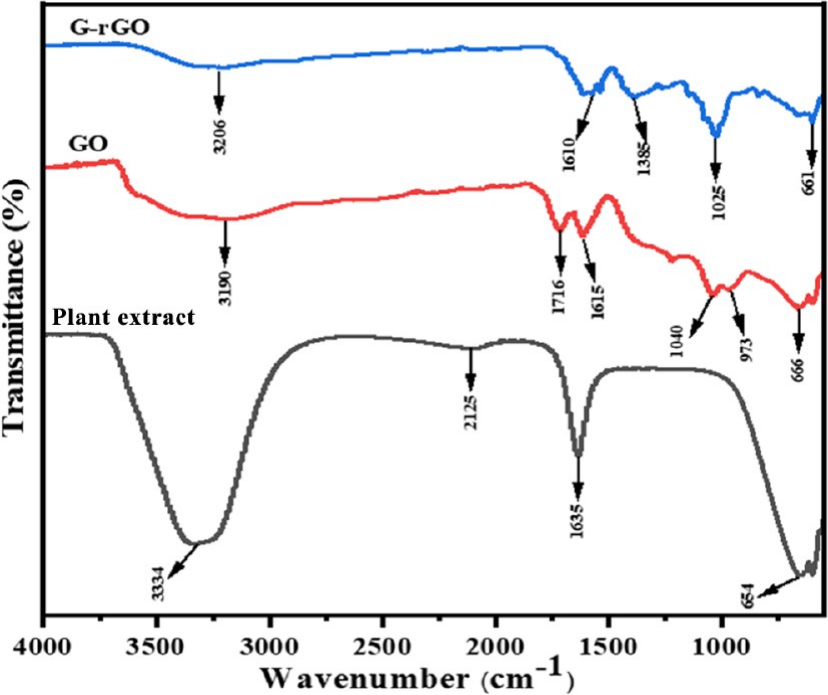
FTIR spectral illustration of the plant extract, GO, and G-rGO from the *T. cordifolia* plant extract.

The FTIR spectrum depicted the functional group transformation of GO before and after reduction and is presented in Figure 3. The C=O stretching vibrations of carbonyl groups were shown strongly in the GO’s FTIR spectra at 1716 cm^–1^^43^ with O–H stretching vibrations and high loading of the hydroxyl (−OH) group at 3190 and 1220 cm^–1^, C=C at 1615 cm^–1^, and C=C bending at 973 and 666 cm^–1^ found in GO’s spectra. In G-rGO, the reduction in the intensity of the peak shows the elimination of groups.^44,45^ The intensities of peaks associated with oxygen functions, such as the C=O stretching peak and the O–H deformation peak, were considerably decreased, implying that graphene oxide has reduced. FTIR analysis confirmed the effective synthesis of graphene oxide and the reduction of graphene oxide (GO). Further, the FTIR spectrum of the *T. cordifolia* plant extract is also depicted in Figure 3. The presence of −OH and –NH stretching is confirmed by the broad peak at 3334 cm^–1^. This overlapped vibration was a result of the presence of amino groups and phenol/carboxylic groups of alkaloids in the extract of *T. cordifolia*.^46,47^ Extremely faint peaks observed at 2125 cm^–1^ represent a C≡C stretching of alkanes. A peak confirmed NH_2_ scissoring and N–H bend of primary amines at 1635 cm^–1^.^47^ The narrow peak at 654 cm^–1^ confirmed the presence of C–Br and C–I stretching.

#### 3.1.3 UV–vis Absorption Spectra of G-rGO

**Figure 4:**
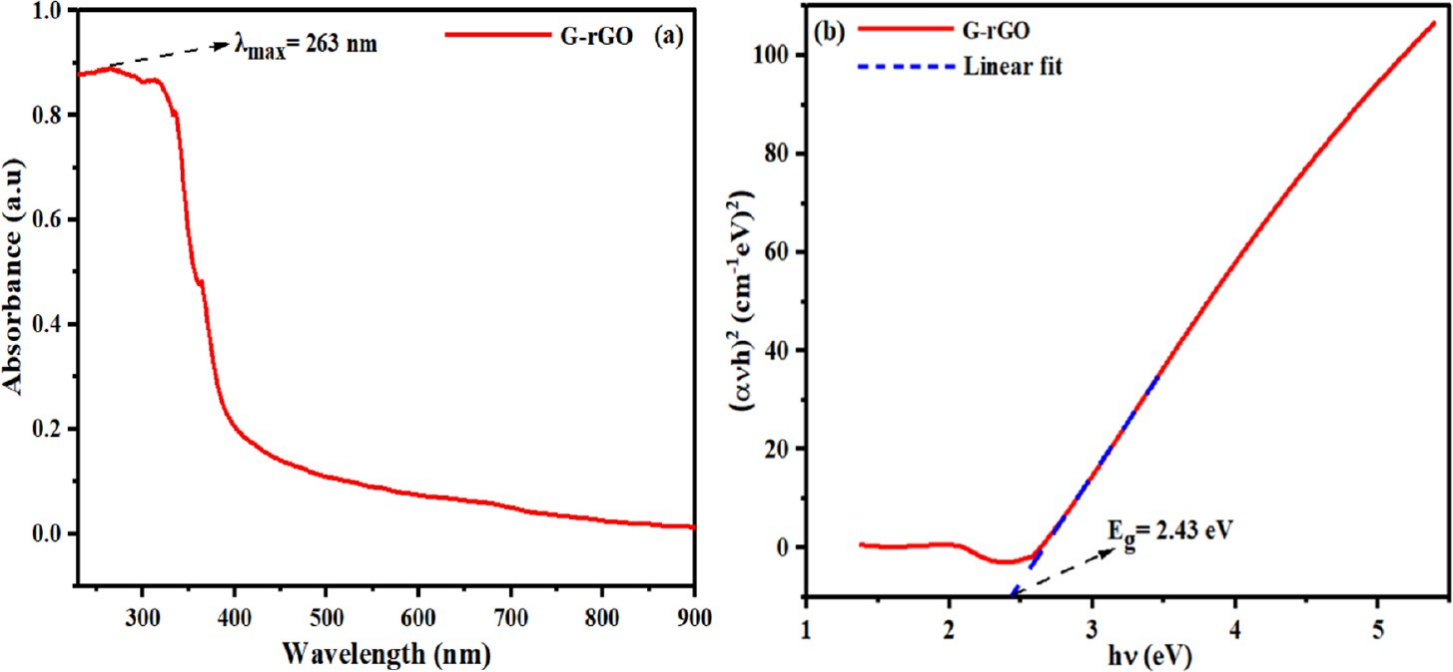
Illustration of (a) absorption spectra of G-rGO and (b) Tauc’s plot for calculation of the energy band gap of G-rGO through linear extrapolation.

The UV–vis spectra of G-rGO show (Figure 4a) that G-rGO has a maximum absorption peak at λ_max_ = 263 nm, indicating the removal of certain groups on the GO surface and that the electronic conjugation has been restored after the reduction of GO.^48,49^ This shift signifies heightened π-electron density and enhanced structural organization, aligning with the revival of sp^2^ carbon and potential atomic reorganization.^50^

The following Tauc’s equation was used to determine the optical band gap energy of the G-rGO based on the UV–vis findings
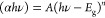
6where α is the absorption coefficient, *h* is the Planck constant (6.626 × 10^–34^ J s), ν represents the frequency of incident light, *A* is a constant, *E*_g_ denotes the optical energy band gap of the semiconductor TiO_2_, and n is 1/2 for direct allowed transitions. The optical band gap energy was calculated from the extrapolation of the linear region to the *x*-axis of the (α*h*ν)^2^ against the h*v* graph (Figure 4b). It was found that G-rGO obtained a 2.43 eV optical energy band gap (*E*_g_). A similar trend was reported for the reduction of the GO using lemon juice as a reducing agent and microwave-irradiated partially reduced GO.^51,52^

#### 3.1.4 ζ Potential and Hydrodynamic Particle Size of GO and G-rGO

**Figure 5:**
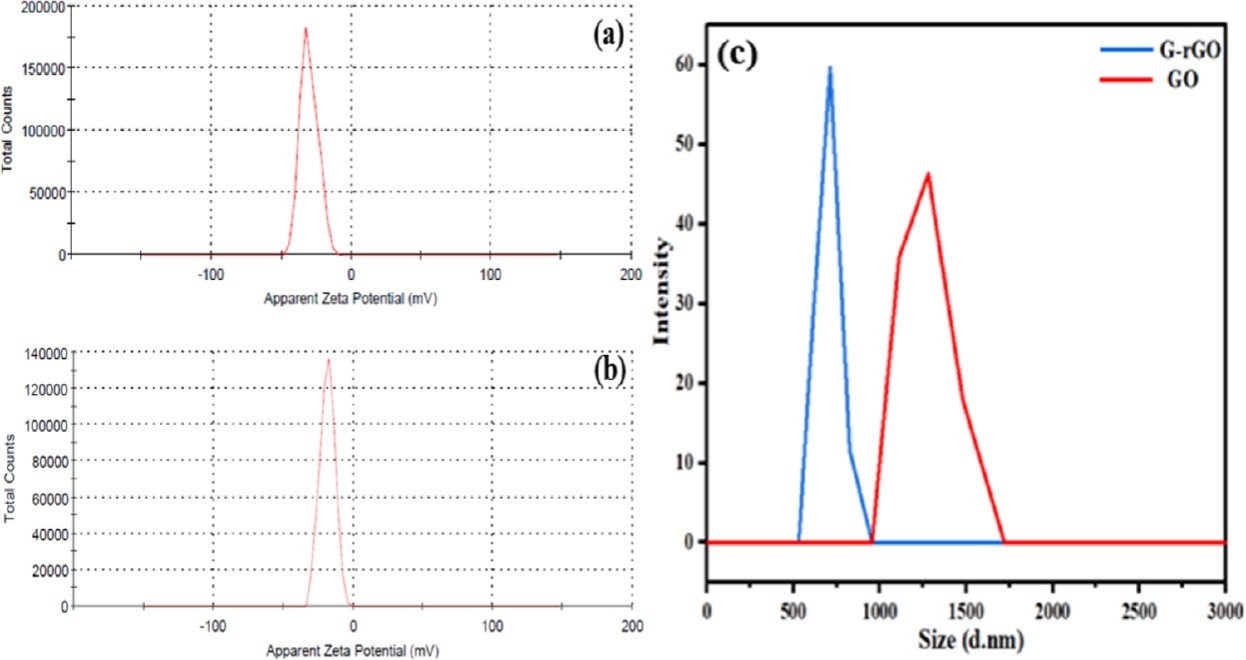
ζ potential distribution according to total counts and intensity: (a) G-rGO and (b) GO. (c) Typical representation of the GO and G-rGO size distribution.

G-rGO has a higher conductivity and is more soluble in water than GO. The ζ potential was used to evaluate the stability of the colloidal dispersion. For solvents in GO- or G-rGO-dispersed solution, the intensity of electrostatic repulsion between similarly charged vicinal sheet surfaces is indicated by the magnitude of the ζ potential. The average value of ζ potential was found to be −29.90 mV for G-rGO, which was higher than 11.50 for GO (ζ = −18.40 mV), demonstrating the increased stability of the G-rGO colloidal dispersion (Figure 5a,b).^53^ G-rGO, with fewer oxygen functional groups and more sp^2^ carbon regions, was formed due to the reduction of GO. The increased sp^2^ domains allowed for stronger π–π stacking interactions between G-rGO and facilitated the formation of a more compact and stable G-rGO structure. The hydrodynamic radius of G-rGO particles was lesser than that of GO.^54^ The range of hydrodynamic radius of G-rGO was 270–470 nm, and for GO, it was 480–850 nm (Figure 5c). The smaller size of a particle means a higher surface area of particles, which was favorable for adsorption.^55^

#### 3.1.5 SEM and EDX Analysis of GO and G-rGO

**Figure 6:**
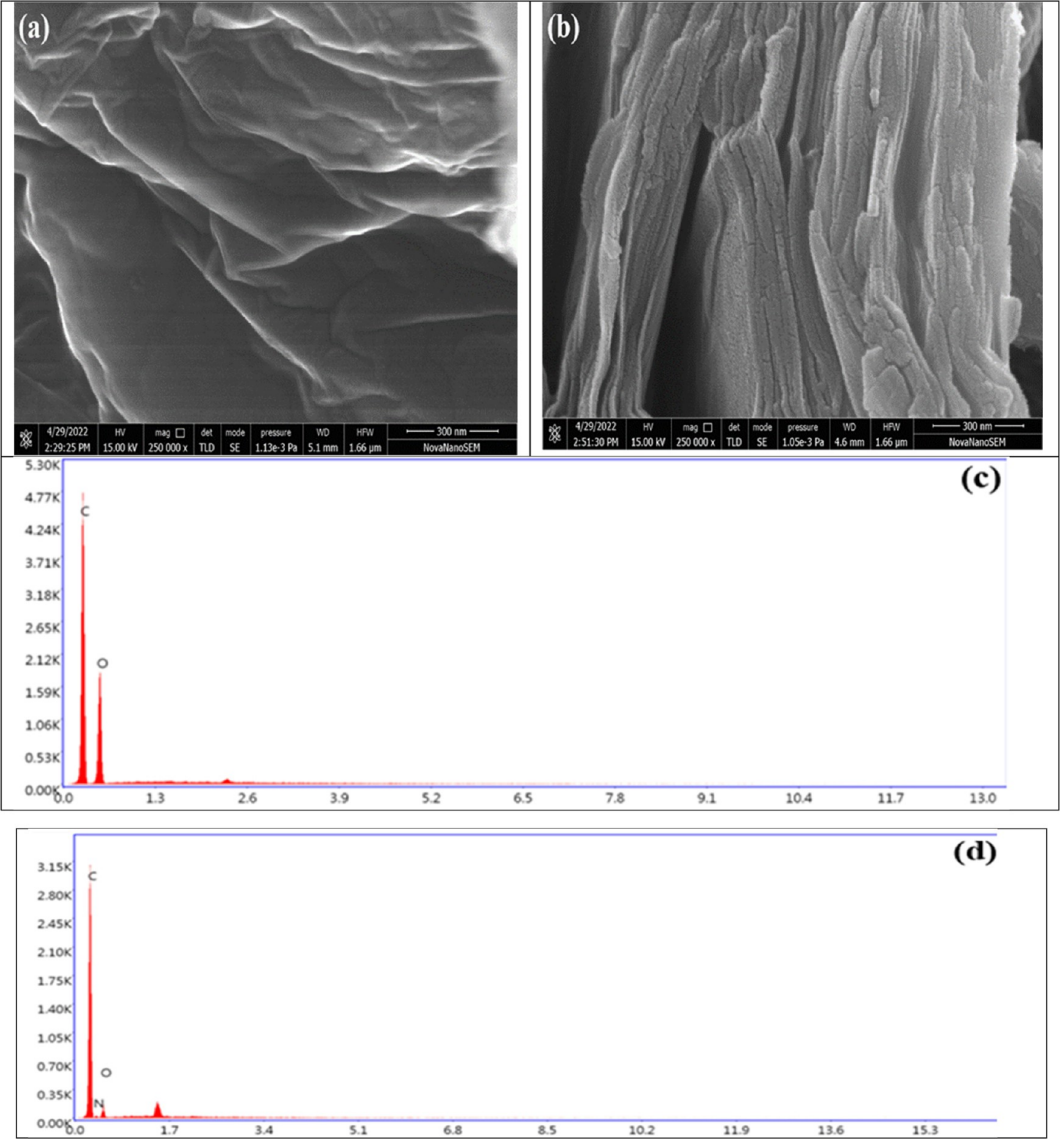
SEM micrographs of (a) GO and (b) G-rGO at ×250 K times magnifications and EDX analysis of (c) GO and (d) G-rGO.

GO and G-rGO’s SEM images are shown in Figure 6a,b. The G-rGO microstructure showed folded and wrinkled sheets (Figure 6b), demonstrating that G-rGO was exploited effectively with less thickness. G-rGO samples had flaky, scale-like structures and resembled closely restacked, undulating silk waves. G-rGO consists of nanosheets with an average width of approximately 30 nm, and the range of the width of G-rGO nanosheets is 20–35 nm. They consisted of discrete, closely interconnected nanosheets, and the outcomes were aligned with resveratrol’s reduction of GO.^56^ The diminished interplanar distance was the result of the elimination of oxygenated groups from the edges and basal planes of G-rGO. This observation was consistent with the XRD results of GO and G-rGO, which showed that G-rGO has a shorter *d*-spacing than that of GO. EDX was used to analyze the elemental compositions of GO and G-rGO (Figure 6c,d). The EDX spectral analysis revealed the atomic percentage of the atomic oxygen present in G-rGO (19.90%) and GO (40.45%) (Table 4). Because oxygen-carrying functional groups had been removed from the surface of GO, the oxygen atomic % had decreased.^57^

**Table 4:** EDX Analysis of GO and G-rGO

Element	GO (wt %)	G-rGO (wt %)
C	59.55	66.37
O	40.45	13.73
N	-	19.90

### 3.2 Estimation of Removal of the MB Dye Using the Response Surface Methodology (RSM)

A batch study was conducted to examine the effects of various parameters on dye removal. As compared to other parameters, dye removal was found to be more influenced by pH, adsorbent dosage, initial concentration of dye, and time.^58^ Therefore, these four parameters were chosen for the present study. A desorption study was additionally carried out, and it was discovered that employing an alkali solution resulted in a higher rate of dye desorption from the adsorbent. The outcomes of every experiment run by software are shown in Table 3. The aforementioned quadratic model describes an empirical relationship between the independent variables and the response.
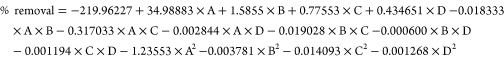
7The findings of the ANOVA analysis confirmed that each parameter contributed to the favored result. The ANOVA statistics for the MB dye adsorption procedure are shown in Table 5. The proposed model’s *F*-value was found to be 183.67, indicating that it was significant. The *p*-value must be less than 0.05, which is analogous to a significant model. In our model, this value was estimated to be less than <0.0001. The model would not be significant if any of the values A (<0.0001), B (<0.0001), C (<0.0001), D (<0.0001), A^2^ (<0.0001), and D^2^ (<0.0001) were larger than 0.1000; however, in this situation, all such values were less than 0.0001. The ANOVA displaying p-values higher than 0.05 (*p* > 0.05) for the interactions AB, AD, and BD suggests that these specific interactions do not exert statistically significant effects on the adsorption process. This could be attributed to several factors: First, the combined effects of variables A and B (AB), A and D (AD), and B and D (BD) may be relatively weak or negligible, indicating that changes in one variable may not significantly impact the adsorption behavior when considered alongside changes in another variable. Second, the observed variability in the response variable, potentially due to experimental noise or uncontrolled factors, may overshadow any effects attributable to these interactions. Third, the study’s sample size and experimental design may lack the sensitivity to detect subtle interaction effects, especially if these effects are small or if the variability in the response variable is high. The values of the constants obtained from both coded and real data are displayed in Table 5. Additionally, the linear regression coefficients (*R*^*2*^), which have a very high value of 0.9942 and indicate an excellent fit between the predicted and actual responses, also define the quality of the regression. Moreover, the predicted *R*^*2*^ (0.9695) and the adjusted *R*^*2*^ (0.9888) were logically consistent (Table 6), demonstrating the model’s ability to predict the process. The signal-to-noise ratio was evaluated using adequate precision. A numerical value of >4 is preferred for modeling. In this study, it was evaluated to be 51.13, suggesting an adequate signal. This model can be applied to navigate the design space.

**Table 5:** ANOVA Analysis of the Quadratic Model (CCD) to Estimate the % of MB Dye Removal

Source	Sum of squares	Df	Mean square	*F*-value	*p*-value	
Model	5864.61	14	418.90	183.67	<0.0001	Significant
A-pH	234.00	1	234.00	102.60	<0.0001	
B-dose	373.51	1	373.51	163.77	<0.0001	
C-initial concentration	617.12	1	617.12	270.59	<0.0001	
D-time	254.41	1	254.41	111.55	<0.0001	
AB	7.56	1	7.56	3.32	0.0886	
AC	2261.48	1	2261.48	991.59	<0.0001	
AD	1.64	1	1.64	0.7184	0.4100	
BC	293.27	1	293.27	128.59	<0.0001	
BD	2.62	1	2.62	1.15	0.3004	
CD	10.40	1	10.40	4.56	0.0496	
A^2^	1635.58	1	1635.58	717.15	<0.0001	
B^2^	19.86	1	19.86	8.71	0.0099	
C^2^	275.77	1	275.77	120.92	<0.0001	
D^2^	180.75	1	180.75	79.25	<0.0001	
residual	34.21	15	2.28			
lack of fit	30.24	10	3.02	3.81	0.0764	not significant
pure error	3.97	5	0.7936			
cor total	5898.82	29				

**Table 6:** Correlation Coefficient for the Proposed Model

*R*^*2*^	0.9942	CV %	1.92
Adjusted *R*^*2*^	0.9888	Mean	78.48
Predicted *R*^*2*^	0.9695	Std. dev.	1.51
Adequate precision	51.1293		

**Figure 7:**
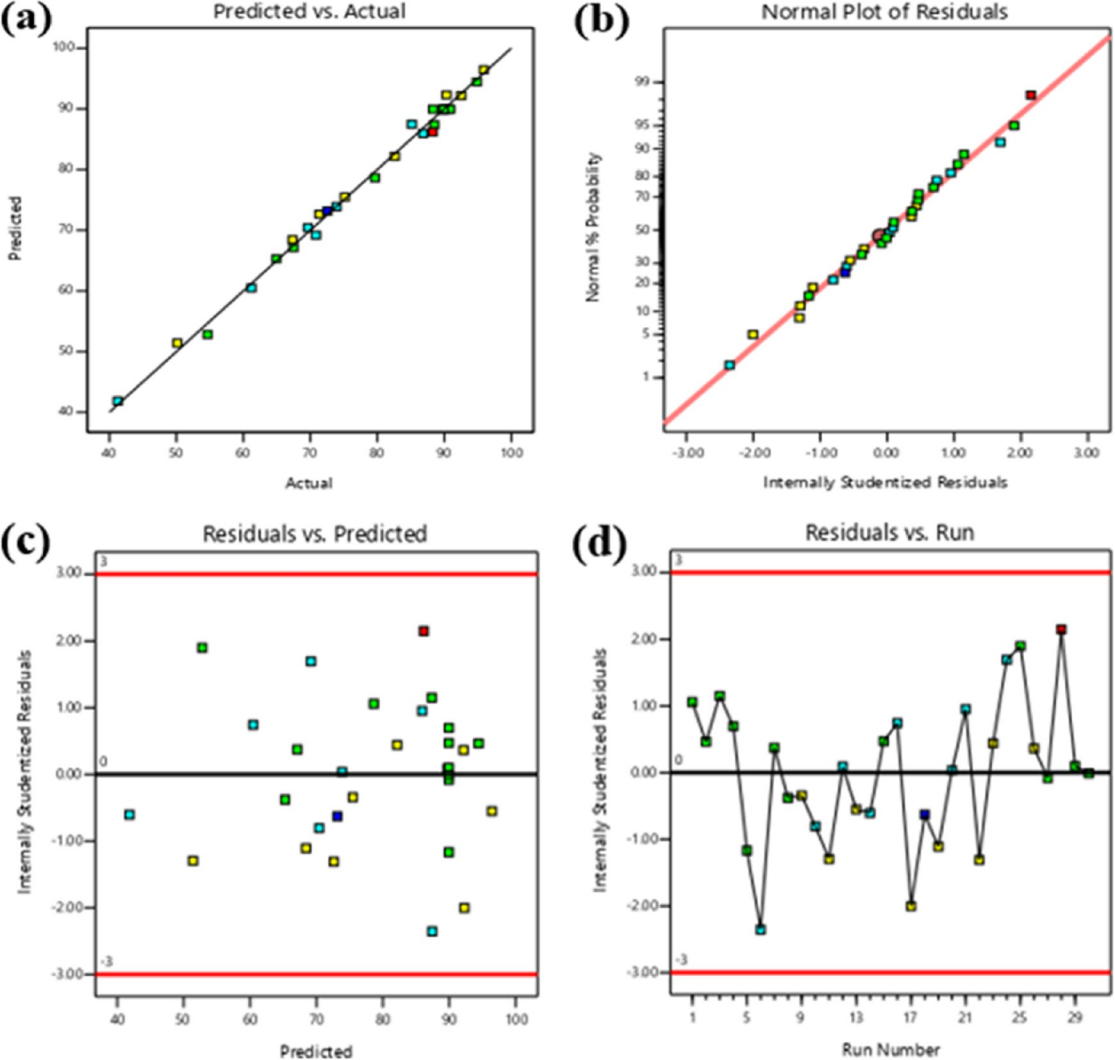
(a) Plot of actual vs predicted % removal of the MB dye. (b) Internally studentized residuals against the normal % probability plot of the % removal of the MB dye. (c) Graph between internally studentized residuals and the predicted % removal of the MB dye. (d) Graph between internally studentized residuals and the run number.

In this case, the coefficient of variance (CV %) and predicted *R*^2^ value were substantially closer. Therefore, it can be concluded that these values validated the model. In Figure 7a, the relationship between the actual and predicted % removal was 1.92%, demonstrating that the standard deviation was lower than the mean. The adjusted *R*^2^ value and MB dye were depicted. The actual experimental response values were substantially closer to the anticipated value of the % removal of MB. This conclusively indicated that the developed model accurately predicted the % removal of MB. Figure 7b presents the standard plot of residuals, which shows the relationship between the internally studentized residuals and normal % probability. It can be concluded that the errors were distributed because residuals were generally scattered along a linear line. The internal studentized residuals and predicted values for the % of MB dye removal are correlated in Figure 7c. The randomly distributed plot demonstrated that for all of the obtained responses, the variance of originally collected data remains the same.^59^ Internally studentized residuals and run numbers for the % removal of MB dye were correlated and are shown in Figure 7d. This plot revealed that there was no valid correlation between experimental data points and the outcomes observed. Design Expert software generated multiple runs for various independent variables. It was evident by examining every parameter that CCD passes every statistical test.^60^

#### 3.2.1 Analysis of Contour Graphs and 3D Surfaces

**Figure 8:**
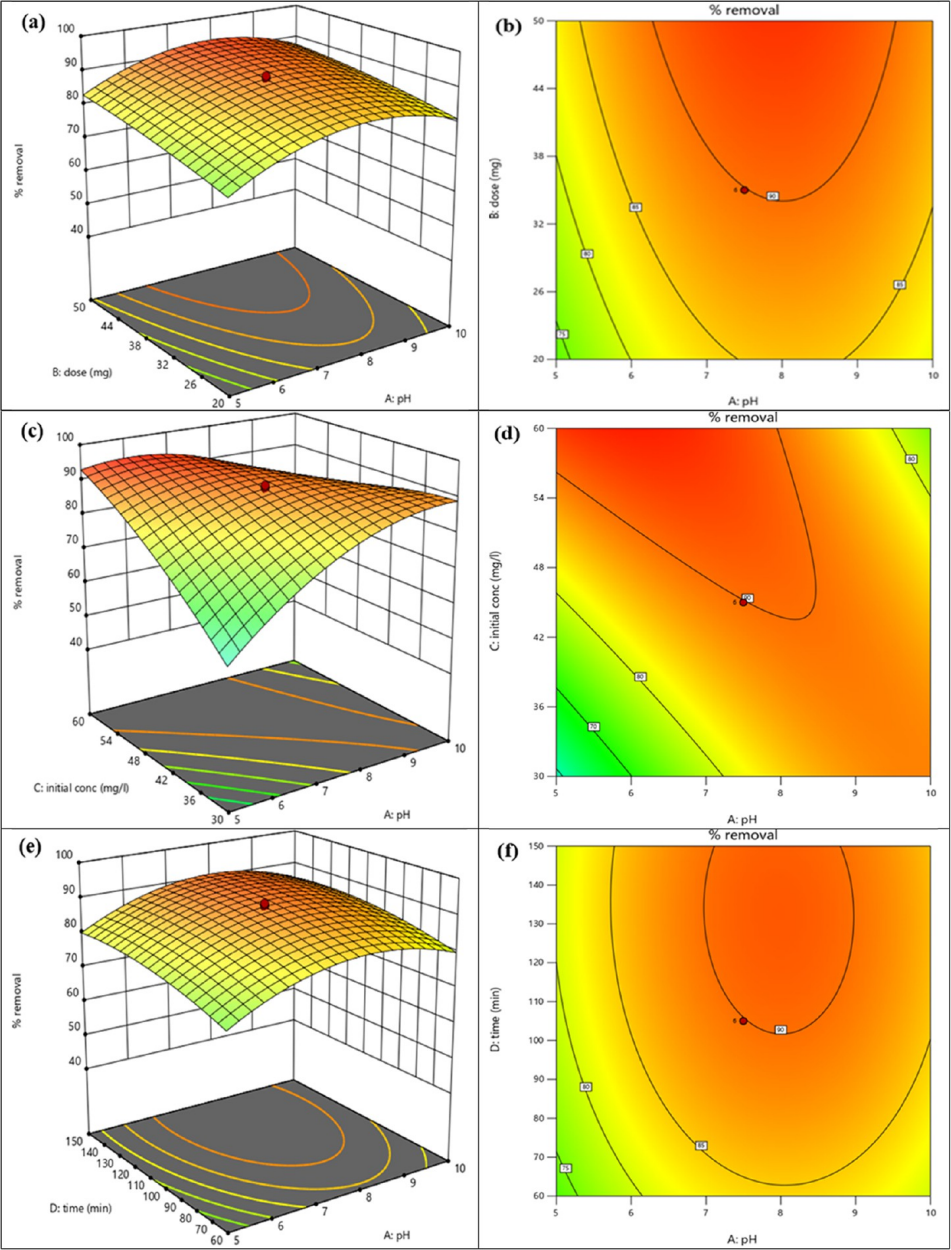
(a) 3D surface illustration of pH (A), adsorbent dose (B), and % removal of MB dye. (b) Contour plot demonstrating the effect of pH (A) and adsorbent dose (B) on % removal of the MB dye, (c) 3D response surface representation of pH (A), initial MB dye concentration (C), and % removal of MB dye. (d) Demonstration using the contour plot between the pH effect (A) and initial concentration of MB dye (C) on % removal of MB dye. (e) 3D surface illustration of pH (A), time (D), and % removal of MB dye. (f) Effect of pH (A) and time (D) on % removal of MB shown by the contour plot.

The selected model for this study was generally represented by 3D curves and contour plots. It provided a precise depiction of the response of the final output in the preferred domain of analysis and the performance of the independent variables. The 3D surface and contour plots demonstrated in Figure 8a,b, respectively, correlated pH (A) and the adsorbent dose (B) with the final response of % removal of the MB dye. According to these figures (Figure 8a,b), at low pH and a high adsorbent dose, the % removal of MB would have the maximum possible value. The interaction between pH (A), initial MB dye concentration (C), and % MB dye removal is illustrated by the 3D surface and contour plot shown in Figure 7c,d. The contour plot and 3D curve revealed that the % removal of the MB dye was highest at low pH and low initial MB dye concentrations. Figure 8e,f depicts the relationship between pH (A), time (D), and % removal of the MB dye. These figures showed that the amount of MB dye removed was greatest at high pH and long duration. Since the functional groups (−OH groups) on the surface of G-rGO deprotonated at high pH, they became more negatively charged, which improved the electrostatic interaction of the MB dye (positively charged) molecules with the G-rGO surface. Longer contact periods also resulted in greater binding between the G-rGO surface and MB molecules due to the chemosorption and stacking interactions that were created between the surface and the MB molecules. Longer contact time allowed MB dye molecules to permeate deeper into G-rGO’s porous surface.^61^

**Figure 9:**
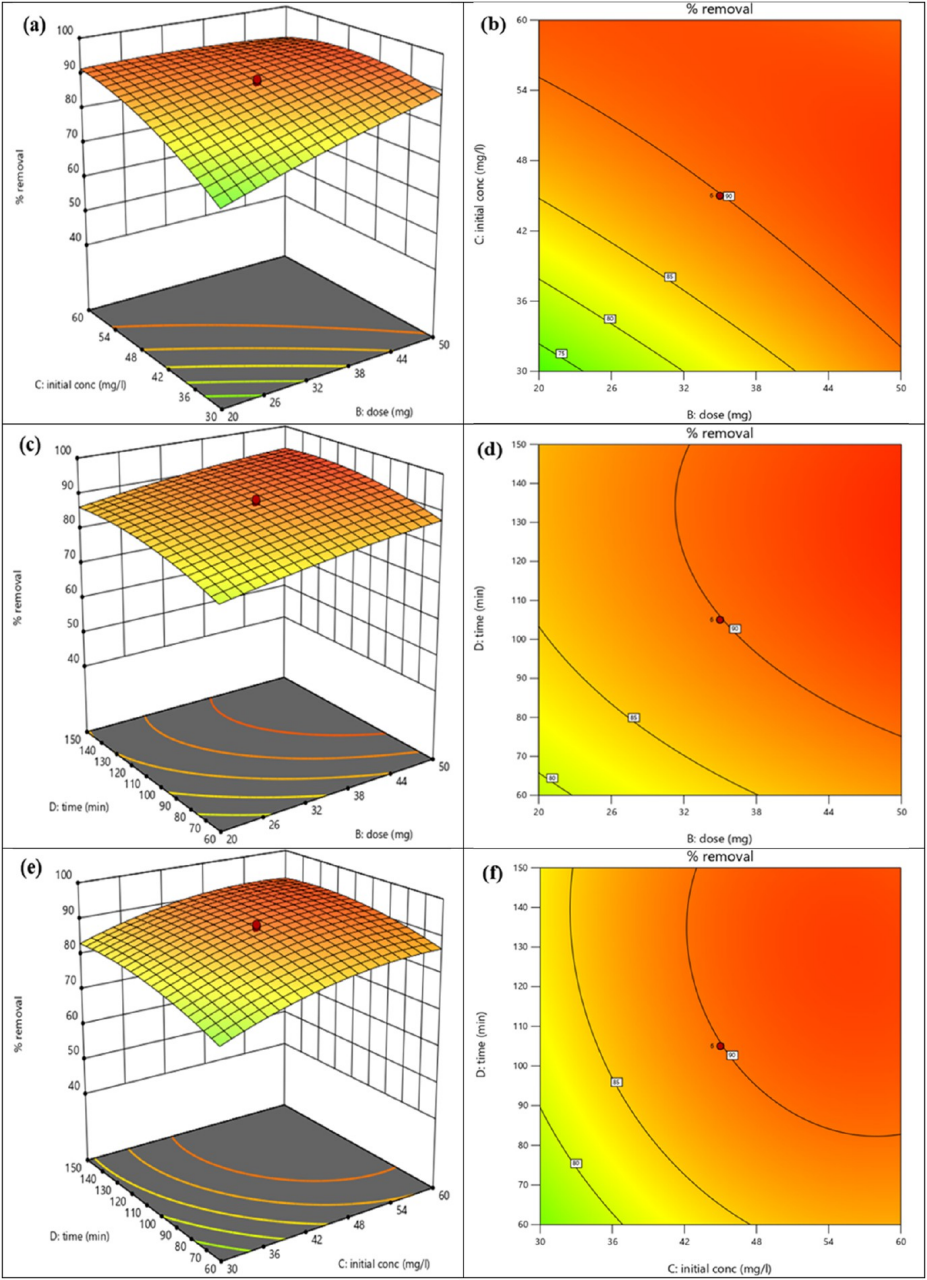
(a) 3D surface illustration of adsorbent dose (B), initial concentration of MB dye (C), and % removal of MB dye, (b) contour plot demonstrating the adsorbent dose effect (B) and initial concentration of MB on % removal of MB, (c) 3D surface representation of adsorbent dose (B), time (D), and % removal of MB dye, (d) contour plot demonstrating the adsorbent dose effect (B) and time (D) on % removal of MB dye, (e) 3D surface illustration of the initial concentration of MB (C), time (D), and % removal of MB dye, and (f) contour plot showing the effect of initial concentration of MB (C) and time (D) on % removal of MB dye.

The 3D surface and the contour plot are demonstrated in Figure 9a,b, respectively, which correlated the adsorbent dose (B) and initial concentration of MB (C) with the % removal of MB dye. These plots proposed that at a high adsorbent dose and low pH, the value of the % removal of MB dye would be maximum. The 3D response surface and the contour curve depict the relationship between the adsorbent dose, time (D), and % removal of MB dye in Figure 9c,d. These plots concluded that the % removal of MB dye was highest at low pH and low initial MB dye concentrations. Figure 9e,f depicts how the initial concentration of MB (C), time (D), and MB dye’s % removal interacted. Both plots concluded that the % removal of MB was highest at higher pH and time.

#### 3.2.2 Optimization Based on the Desirability Function

**Figure 10:**
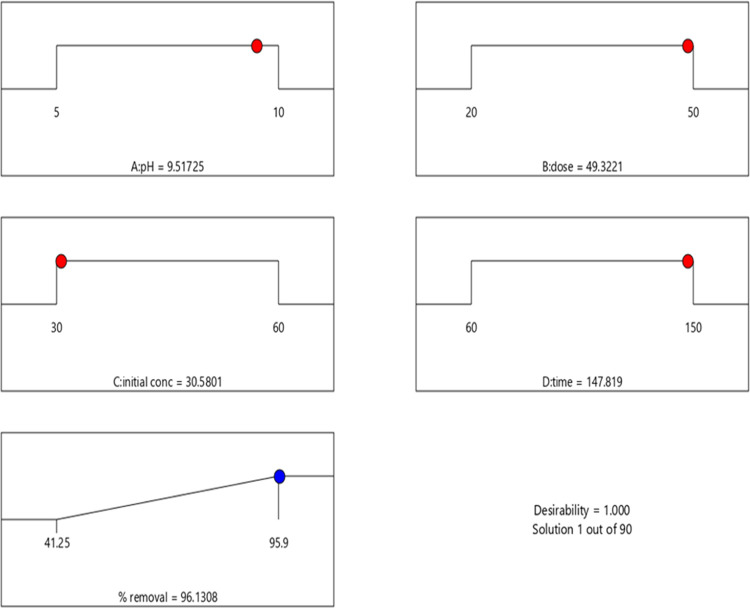
Desirability ramp for numerical optimization of objectives: initial pH of the solution, adsorbent dose, initial concentration of dye, time, and % removal of MB dye.

The desired outcomes for response and each variable were elected in numerical optimization from design software. The potential objectives for response were to maximize, target within the range, minimize, none, and specify an exact value for the factors. For every mentioned parameter, the levels must be defined (minimum or maximum). Each objective can be given a weight to modify the specific desirability function’s shape. A total desirability function was created by combining the objectives. The desirability function has a range of 0 to 1, 0 for outside limits and 1 for the goal. The attempt to achieve the goals initiates at an arbitrary point and proceeds until it reaches its peak. Due to the curvature in response surfaces, multiple maxima may exist and how they interact with the desirability function is studied. Finding the ’best’ local maximum seems to have a greater tendency when starting from plenty of points in the design space. A multiple-response method was used to optimize any combination of objectives, including the initial MB concentration and pH of the solution, adsorbent dose, time needed, and % of MB removal. Through numerical optimization, optimum points were used to generate ramp desirability (Figure 10). The optimum local maximum was discovered by observing the response surface changes from the starting points to be at an adsorbent dose of 49.32 mg, initial concentration of 30.58 mg L^–1^, initial solution pH of 9.52, time of 147.82 min, and MB dye removal of 96.13%. The temperature and agitation speed were 30 ± 5 °C and 130 rpm, respectively. The obtained desirability value demonstrated that the anticipated function might adequately represent the desired conditions and experimental model.

#### 3.2.3 Confirmation Experiments

**Figure 11:**
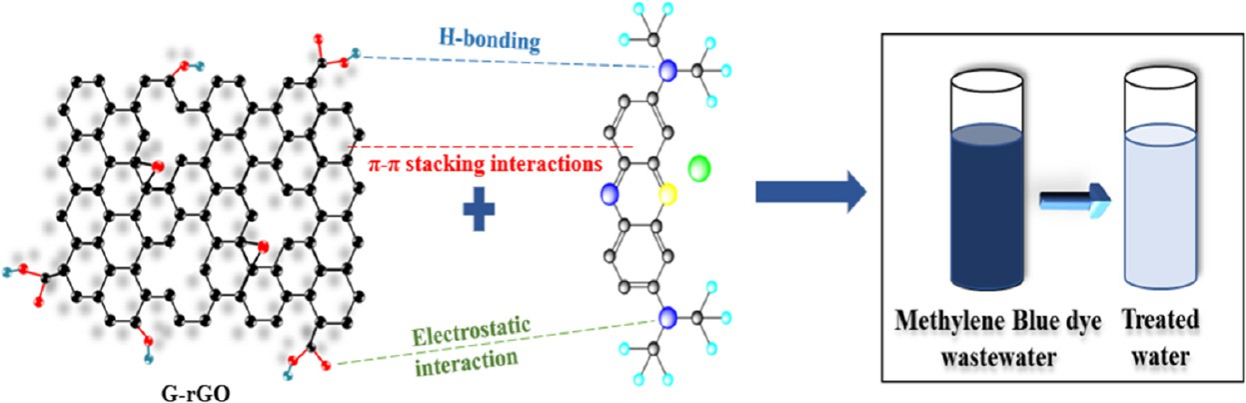
Schematic representation of the adsorption mechanism of MB dye degradation by G-rGO.

Experiments were performed to validate the parameters predicted by the model (pH 9.52, initial MB concentration of 30.58 mg L^–1^, time of 148 min, adsorbent dose of 49.32 mg, agitation speed of 130 rpm, and temperature of 30 ± 5 °C) to support the predicted data generated through numerical modeling at optimized conditions. MB dye molecules in solution adsorb on G-rGO sheets through π–π stacking and electrostatic interactions. The surface area and conductivity of G-rGO enhance the breakdown of adsorbed molecules. Electron transfer from G-rGO reduces MB and converts it into less harmful compounds. G-rGO generates ROS, which aid in degradation through oxidative processes (Figure 11). The MB dye removal was found to be 94.85% with a deviation of 1.28%. It has been observed that there was a relatively minor deviation between the predicted % removal and actual % removal. Effective experimental design, validation of models, and a comprehensive understanding of the system’s complexities are needed to enhance the accuracy of RSM predictions. The adsorption capacity at the corresponding optimum conditions was found to be 58.81 mg g^–1^.

### 3.3 Adsorption Isotherm

**Figure 12:**
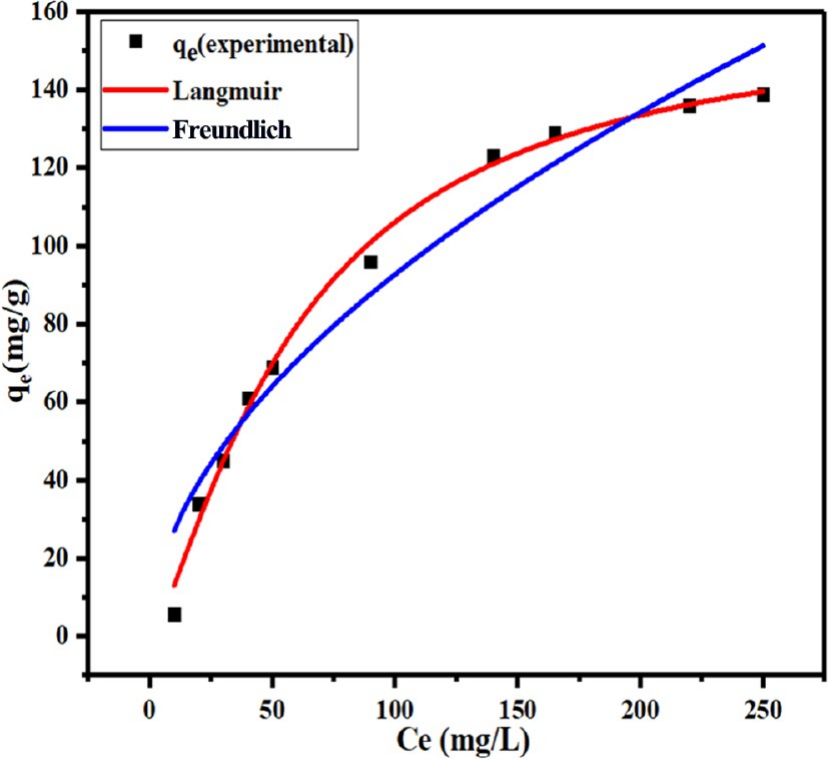
Evaluation of the stability of Freundlich and Langmuir isotherm models to explain MB dye adsorption onto G-rGO.

The suitable isotherm model shows adsorption capacity and how adsorption molecules have interacted between the adsorbent and adsorbate at an equilibrium state in adsorption. Langmuir and Freundlich isotherm models are the most commonly used isotherms to represent the experimental data of adsorption. Both isotherms were used to study the adsorption of MB with G-rGO (Figure 12). The adsorption capacity exhibited an increasing trend, eventually reaching a plateau at its maximum. To determine the type of isotherm, the value of 1/n can be used in the Freundlich isotherm. The isotherm will be irreversible (1/*n* = 0), undesirable (1/*n* > 1), and desirable (0 > 1/*n* < 1), and at *n* = 1, adsorption linearly decreases. In this study, the 1/*n* value of the Freundlich isotherm was 0.53, which shows an ideal adsorption performance.^62^ The Langmuir model showed better fitting with experimental findings (*R*^2^ = 0.9928), which implies that MB dye adsorption on G-rGO occurred as a monolayer and at homogeneous sites.^63^ The parameters of models were calculated for both isotherms and are shown in Table 7. According to the above finding, the G-rGO had a homogeneous surface and the MB dye was evenly entrapped in the surface.

**Table 7:** Langmuir and Freundlich Isotherm Model Parameters

S.N.	Langmuir isotherm parameters	Freundlich isotherm parameters
1.	*Q*_m_ = 160.45 mg/g	*K*_f_ = 7.92 L/g
2.	*K*_l_ = 0.004 L/g	*n* = 1.87
3.	*R*^2^ = 0.9928	*R*^2^ = 0.9650

### 3.4 Kinetic Study

**Figure 13:**
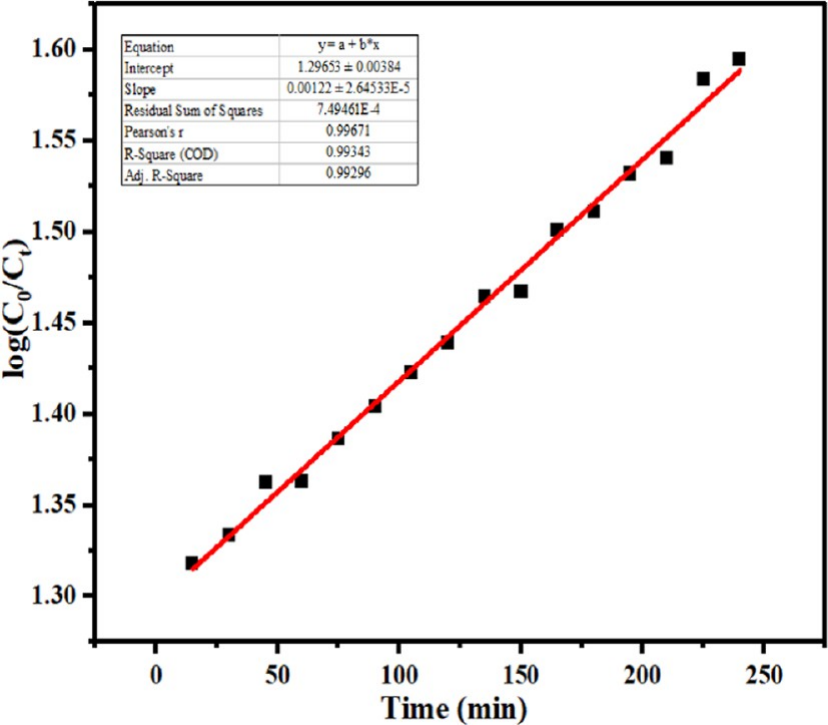
Kinetics of adsorption of MB on G-rGO, linear fitted graph between ln  and time.

The adsorption kinetics was conducted concerning time. A UV–visible spectrophotometer was used to measure the absorbance of fractions of samples taken out at regular intervals as the reaction proceeded. The pseudo-first-order kinetic model assumes that the rate of adsorption is directly proportional to the number of available adsorption sites on the adsorbent surface, which is often the case for G-rGO materials with a high surface area and abundant π-electron systems. The kinetic plot between ln  and time was used to calculate the reaction’s rate constant (Figure 13). It was found that the adsorption process of the MB dye on G-rGO was typically rapid initially, with a large number of dye molecules rapidly adsorbed onto the surface of the graphene. This initial rapid adsorption phase aligns well with the assumptions of the pseudo-first-order kinetic model. It was also found that the pseudo-first-order possesses the *R*^2^ value of 0.99343, which means that the pseudo-first-order model closely predicts the adsorption behavior of MB on G-rGO.
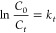
8In this equation, *C*_0_ and *C_t_* are the MB dye concentration at the initial and final conditions, respectively, and *k* is the reaction’s rate constant.

The reaction rate constant of adsorption of the MB dye on G-rGO was determined from the slope of the graph between ln  and time, and it was found to be *k* = 0.00122 min^–1^.

### 3.5 Recycling Test of the Adsorbent

**Figure 14:**
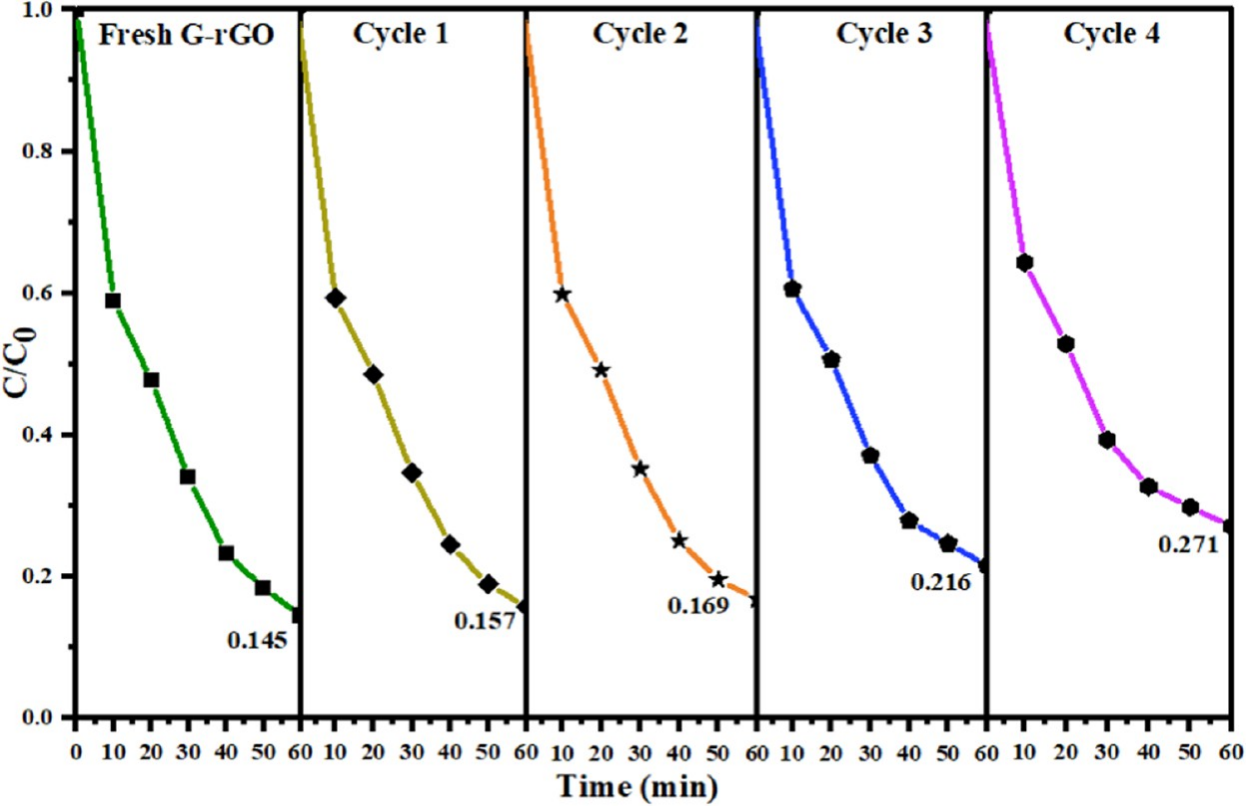
Recycling test of the MB dye by G-rGO (initial MB concentration: 30 mg L^–1^, adsorbent dose: 50 mg, pH: 9, agitation speed: 130 rpm, and temperature: 30 ± 5 °C).

Recycling the adsorbent after adsorption is an important and cost-effective procedure in wastewater treatment. The adsorbent must have high adsorption as well as good desorption capabilities to reduce the cost of adsorption significantly. Therefore, the regeneration and reuse of G-rGO were examined by separating G-rGO after the adsorption of the MB dye (at optimum conditions for 60 min adsorption duration) using centrifugation (300 rpm). The centrifuged adsorbent was added to the ethanol solution, and the mixture was stirred for 15 min. Then, it was filtered (Whatman 41 filter), rinsed with double-distilled water several times, and then dried at 70 °C. The same procedure was conducted four times to prove the efficient recovery and reuse of G-rGO.^64^ It was found (Figure 14) that a 14.74% decrease was observed in the % removal of MB dye after fresh to the fourth recycle of G-rGO (85.52–72.89%).

### 3.6 Antibacterial Activity of G-rGO

**Figure 15:**
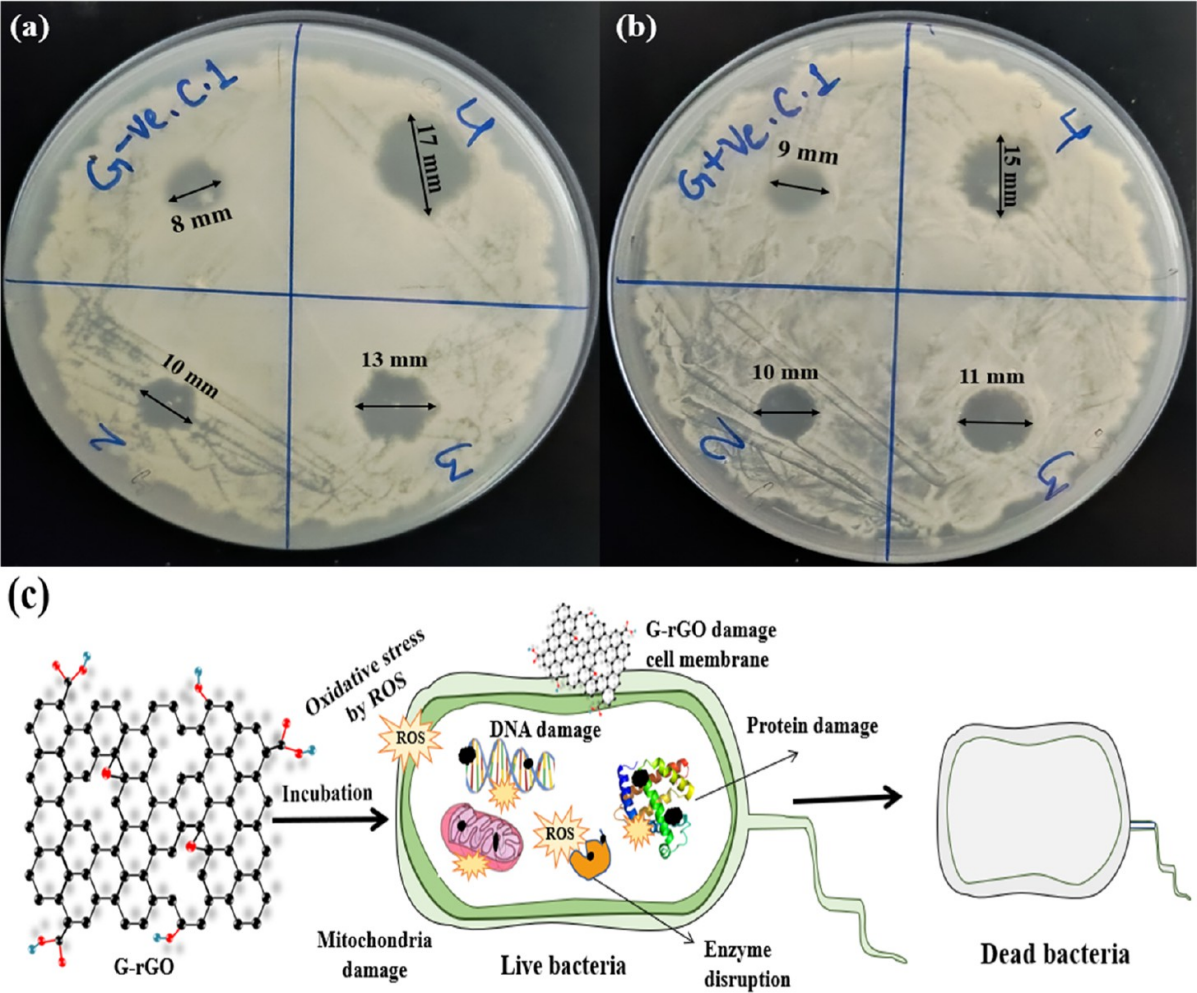
Petri dish shows the antibacterial potential of G-rGO against (a) *E. coli* (Gram-negative) bacteria and (b) *Staphylococcus aureus* (Gram-positive) bacteria and (c) schematic representation of the mechanism for antibacterial activity of G-rGO against bacteria.

*E. coli* (Gram-negative) bacteria and *Staphylococcus aureus* (Gram-positive) bacteria were used to investigate the antibacterial activity of G-rGO. *E. coli* and *S. aureus* have a spherical shape and smaller size. The growth of *S. aureus* and *E. coli* was diminished at different marked places on the Petri dishes having different amounts of G-rGO. It was observed that at 20, 40, and 80 μg mL^–1^ concentrations of G-rGO with 20 μg mL^–1^ GO as a control incubated in a Petri dish at 30 ± 5 °C for 12 h, the zone of inhibition was measured to be approximately 10, 13, and 17 mm, respectively, and 8 mm for the control (Figure 15a) against *E. coli* Gram-negative bacteria. Similarly, against *S. aureus*, the zone of inhibition was measured to be approximately 10, 11, and 15 mm, respectively, and 9 mm for the control (Figure 15b). The primary mechanism by which G-rGO destroys bacterial cells is by mechanically stressing the cell membrane.^45^ Reduction of GO to rGO restores the sp^2^ carbon network and reduces the number of oxygen-containing functional groups, resulting in a more hydrophobic and biocompatible surface. This alteration in surface chemistry promotes stronger interactions between rGO and bacterial cell membranes, facilitating membrane disruption and increased penetration of rGO into bacterial cells. Additionally, the improved electrical conductivity of rGO enables efficient electron transfer processes, leading to oxidative stress and damage to bacterial DNA and proteins. Furthermore, the larger surface area and enhanced π–π stacking interactions of rGO enhance its ability to adsorb and immobilize bacterial cells, further contributing to its antibacterial efficacy (Figure 15c). It shows the antibacterial property of G-rGO against Gram-negative (*E. coli*) bacteria^65^ as well as against Gram-positive (*S. aureus*) bacteria.^66^

## 4 Conclusions

The present work emphasizes the first-ever green production of G-rGO using the naturally available *T. cordifolia* plant extract by reduction of GO. A modified Hummer’s method was used to synthesize GO. The feed and extract were characterized using different analytical tools. Also, the RSM was used to examine the combined impact of several process parameters on MB dye removal. The HD-XRD results confirmed an increased crystallinity of G-rGO, and FTIR confirmed an improved function group. The adsorption study of MB confirmed that under optimum conditions, the removal efficiency of MB was determined to be 94.85%, with an adsorption capacity of 58.81 mg g^–1^. The kinetics of adsorption linearly followed the pseudo-first order and obtained 0.00122 min^–1^ rate constant of the reaction. Finally, the typical antibacterial activity was studied, and the findings supported the G-rGO’s antibacterial activity against *S. aureus* and *E. coli*. Therefore, the present study finding exhibited green synthesis as an innovative, economical, and environmentally friendly approach to the formation of G-rGO and its excellent antibacterial activity.

## Data Availability

The datasets generated during and analyzed during the current study are available from the corresponding author upon reasonable request. We have chosen not to make the data supporting our paper publicly available due to privacy restrictions. This decision is in accordance with our institution’s policies and ensures the confidentiality and privacy of data.
